# Assessing the Safety and Efficacy of a Noninvasive Device in the Management of Musculoskeletal Pain Using Low-Level Light Therapy: Double-Blinded, Randomized, Placebo-Controlled, Multicentric Study

**DOI:** 10.2196/87566

**Published:** 2026-04-16

**Authors:** Surya Prakash Maguluri, Vinod Kumar Penubakala, Sai Laxman Anne, Shankar A Navaladi, Chandrashekar P, Antony Leo Aseer, Ashok Kumar B K, Mohamed Sameer M, Radhika Soanker, Vikas Sharma

**Affiliations:** 1 Litemed India Private Limited, Plot No 8, Flat No 301, Saikrupa Enclave, Sairam View, opposite Chicken Basket, near Ghanshyam Supermarket, Khajaguda, Rangareddi, Hyderabad, Telangana, 500089, India, 91 79810 29973; 2 Department of Orthopaedics, Krishna Institute of Medical Science Enterprises Limited, Hyderabad, Telangana, India; 3 Department of Orthopaedics, Apollo Hospital Enterprise Limited, Chennai, Tamil Nadu, India; 4 Department of Orthopaedics, Nizam's Institute of Medical Sciences, Hyderabad, Telangana, India; 5 Department of Physiotherapy, Sri Ramachandra Institute of Higher Education and Research, Chennai, Tamil Nadu, India; 6 Department of Pain Management, Kempegowda Institute of Medical Sciences, Bangalore, Karnataka, India; 7 Department of Orthopaedics, Sri Ramachandra Institute of Higher Education and Research, Chennai, Tamil Nadu, India; 8 Department of Pharmacology, All India Institute of Medical Sciences, Bibinagar, Hyderabad, Telangana, India

**Keywords:** photobiomodulation, pain management device, pain, low-level light therapy, infrared therapy

## Abstract

**Background:**

Musculoskeletal pain significantly impacts quality of life and daily functioning. Light-based therapies, including those using red and infrared wavelengths, have shown potential in pain management due to their anti-inflammatory and tissue healing properties. CURAPOD, a pain management device developed by Litemed, uses a combination of visible red and infrared light for noninvasive pain relief.

**Objective:**

This study aimed to assess the safety and efficacy of Litemed’s pain management device (CURAPOD) in managing acute and chronic musculoskeletal pain at various pain sites, in comparison with a placebo, and to evaluate its efficacy across different skin types.

**Methods:**

In a double-blinded, randomized, placebo-controlled, multicentric study, 240 participants (aged 18‐60 years) with acute or chronic musculoskeletal pain were enrolled and treated with either the test or control device for 30 minutes. The test device contains 7 LEDs designed to emit a combination of visible red and infrared radiation, while the control device emits visible light of the red spectrum. Pain intensity was subjectively measured at baseline, at 30 minutes after treatment, and at time windows of 8 to 12 hours and 21 to 24 hours after treatment.

**Results:**

A greater number of participants reported a reduction in pain (of up to 60%) in the treatment group than in the placebo group. Repeated measures ANOVA revealed significant effects of time (*F*_3,684_=282.37; *P*<.001) and treatment group (*F*_1,228_=662.12; *P*<.001), indicating that the relief experienced may be more sustained in the treatment group. No significant effects of pain site (*F*_5,228_=0.169; *P*=.97) or skin type (*F*_5,228_=0.8; *P*=.55) were observed, suggesting consistent action across anatomical locations and skin types. No significant adverse events were reported.

**Conclusions:**

The device appears to be safe and viable as a nonpharmacological adjunct for managing acute and chronic musculoskeletal pain. Treatment with the device showed short-term pain reduction, with reports of up to 60% pain reduction within 30 minutes of use and sustained self-reported relief in pain for up to 20 hours after treatment. No significant effects of pain sites or skin type on reduction in visual analog scale scores were observed, suggesting broad applicability. However, these results must be interpreted with caution while considering the limitations inherent to the study methodology and short-term follow-up. These findings should be interpreted as evidence of short-term analgesic response rather than definitive clinical effectiveness. Further investigation through rigorously designed randomized controlled trials and longitudinal studies is necessary to definitively establish the efficacy of the device, the durability of its action, and the potential integration of CURAPOD into pain management strategies.

## Introduction

Pain is believed to be the most universally recognized and oldest symptom of ill health for which people seek medical attention. Millions of patients approach medical facilities for pain-related issues, regardless of age or gender [1].

According to the Global Burden of Disease assessment (2019), musculoskeletal conditions are the most prevalent globally, with lower back pain as the leading contributor. An estimated 1.7 billion people are reported to be affected by musculoskeletal conditions [2], with pain associated with these conditions being the most common form of noncancer pain [3].

Pain limits the ability to accomplish daily and social activities, leading to a cascade of psychological problems such as anxiety, depression, sleep disturbances, impaired cognition, lack of focus, and decreased productivity, which presents a significant burden to the health care system and global economy. The International Association for the Study of Pain has been reinforcing the notion that pain should be recognized as a disease and not merely a symptom [4,5].

The most popular and widely accepted approaches to the management of pain are the use of opioid and nonopioid analgesics (nonsteroidal anti-inflammatory drugs and acetaminophen) with adjuvants (anticonvulsants and antidepressants), which could potentially cause undesirable drug interactions, adverse drug reactions, and a multitude of short- and long-term side effects [6].

Opioids have a known propensity to induce drug tolerance; dependence; and, paradoxically, hyperalgesia when used for extended periods. Their potential for drug dependence and abuse is a significant factor that contributes to the global opioid crisis [7,8].

Nonnarcotic analgesics, such as nonsteroidal anti-inflammatory drugs and acetaminophen, are generally safer and more readily available. However, they can still cause side effects, including gastritis; an increased risk of internal bleeding; and, with chronic use, liver and kidney toxicity [9].

The decisional quandary in pain management lies in the choice of interventions for pain relief to improve quality of life [8], which can be addressed by a multimodal pain management plan emphasizing patient education and self-management strategies that are beneficial to reducing dependence on medication and attenuating their side effects [10]. The biopsychosocial model recognizes that pain is a multifaceted problem with biological, psychological, and social factors [11], offering a holistic approach to pain reduction that combines the use of pharmacologic analgesics in conjunction with psychosocial interventions, such as stress reduction; cognitive behavior therapy; and lifestyle changes with physical therapeutic modalities, including acupuncture, cryotherapy, transcutaneous electrical nerve stimulation, heat therapy, and low-level light therapy [6,10,12].

Low-level light therapy, or photobiomodulation, uses nonthermal, nonionizing light therapy to facilitate tissue healing and was discovered by Hungarian physician Endre Mester in 1967 [13]. It has since been researched widely to elucidate that the primary effects of photobiomodulation occur during irradiation and are mediated by cytochrome c oxidase, a chromophore present within the mitochondrial matrix [14]. As an integral part of the electron transport chain, cytochrome c oxidase, when activated, increases the oxygen intake of the cell and the production of adenosine triphosphate, altering the intermembrane potential and regulating reactive oxygen species. Subsequently, a cascade of secondary effects includes regulation of inflammatory cytokines and gene expression to alter signaling pathways and transcription factors [15,16]. Additionally, there is an increase in the availability of nitric oxide, which may be due to the effects on the mitochondria and direct photodissociation, resulting in an increase in blood flow to the tissues [17]. These processes collectively aid in tissue healing and pain relief.

Lasers were initially used to deliver low-power, high-fluence monochromatic light for therapeutic purposes, which led to the term low-level laser therapy. Later, LEDs were found to produce similar effects, leading to the more encompassing photobiomodulation [14]. LEDs do not produce coherent light, unlike their laser counterparts, which help them achieve their therapeutic effect with a lower risk of burns that are usually associated with laser therapy [18]. Litemed’s CURAPOD device is a noninvasive, compact, portable device that uses these principles of photobiomodulation to deliver a combination of therapeutic wavelengths of light through LEDs, allowing it to be self-administrable. With little to no side effects, the device aims to provide a safe and effective alternative or complement to traditional pain management practices that often carry documented risks. This study evaluated the effectiveness of CURAPOD therapy for pain relief across various anatomical pain sites among patients with different skin types.

## Methods

This was a randomized, double-blinded, multicentric, 2-arm, parallel, placebo-controlled study conducted with a study population of 240 participants.

### Ethical Considerations

This study was conducted in accordance with the ethical guidelines of the current version of the Declaration of Helsinki (64th World Medical Association General Assembly, Fortaleza, Brazil, October 2013). The trial was conducted in accordance with the International Conference on Harmonisation guidelines on Good Clinical Practice E6 (R2).

Ethics approval was obtained at each site from its respective institutional ethics committee.

Ethics approval for the final study protocol was obtained prior to study initiation from the following:

- The Institutional Ethics Committee for Biomedical Research, Apollo Hospital, Chennai (AMH-C-S-027/05‐22 approved on May 20, 2022)
- The Sri Ramachandra Institutional Ethics Committee, Chennai (IEC/22/APR/171/32 approved on August 3, 2022)
- The Nizam’s Institute of Medical Sciences Institutional Ethics Committee, Hyderabad (EC/NIMS/3045/2022 approved on September 8, 2022)
- The KIMS Ethics Committee, Hyderabad (KIMS/EC/2022/95‐03 approved on September 9, 2022)

All participating centers initiated the study only after receiving the respective ethics committee approvals.

Written informed consent was obtained from all participants prior to the performance of any study-related procedures, following which only anonymized, deidentified participant data were used for the purpose of the study, and appropriate measures were taken to ensure participant confidentiality throughout the study. Participants were not compensated for their role in the study in compliance with Indian Council of Medical Research’s ethical guidelines.

### Study Objectives and Design

Our objective was to evaluate the safety and efficacy of CURAPOD as an adjunct for managing acute and chronic pain arising from various sites in the body. Additionally, the efficacy of CURAPOD across different skin types defined by the Fitzpatrick scale (I-VI), which classifies skin by UV response, was assessed, along with the time to recurrence of pain following a single treatment session [19].

The study was conducted at 4 tertiary care hospitals across South India over a 3-month period, during which individual patient participation lasted 1 visit, with telephonic follow-up at 8 to 12 hours and 21 to 24 hours after treatment. A sample size of 240 participants, randomized in a 1:1 allocation ratio, with 120 (50%) participants assigned to each treatment group, was calculated assuming a minimum mean difference of 1 point in the change from baseline visual analog scale (VAS) score between the test and placebo groups over 1 day, an assumed SD of 2 points, 85% statistical power, a 2-sided α level of .05, and an anticipated dropout rate of 20%.

Adults aged 18 to 60 years were screened and enrolled based on a clinical diagnosis of either acute or chronic pain of musculoskeletal origin, as determined by the site investigator (based on medical history and physical examination), with an intensity on the VAS ranging from 4 to 7 on a scale of 0 to 10 [20].

For diagnosis, acute pain was defined as pain of short duration, typically lasting less than 3 months and associated with a healing tissue injury. Chronic pain was characterized by its persistence beyond the normal healing period, generally extending beyond 3 months. Patients with pain localized to the neck, shoulder, back, arm, knee, and leg were considered, and they were required to abstain from seeking any other pain relief treatments during the study.

Patients with psychosomatic pain conditions, deep vein thrombosis, varicose veins, gout, ganglionic cysts, bone fractures, cervical spondylosis, and postoperative surgical pain were excluded from the study. Patients with pain intensity greater than 7 on the VAS scale were also excluded to minimize patient discomfort. Additionally, those with unhealed injuries that resulted in a break in the continuity of the skin at the pain site were also excluded, as it may lead to complexities in device application.

Anyone with a known drug allergy to aceclofenac and diclofenac (planned rescue medication for the study) was also excluded, as were those who had received any other pain management treatment 24 hours prior to their enrollment.

### Randomization and Blinding

The study population was divided into 2 arms of 120 participants each, one receiving treatment with the experimental intervention and the other with a placebo. SAS software (version 9.4; SAS Institute Inc) was used to allot participants into their treatment groups in a 1:1 ratio using stratified block randomization, with the 6 pain sites forming the strata. Knowledge of randomization was restricted to those responsible for generating it. A sealed copy of the randomization scheme and code was retained at the study site. It could be accessed in case of emergency unblinding to verify the treatment identity of individual subjects.

The investigational product and placebo were labeled and packaged at the manufacturing site to ensure blinding. An independent dispenser, who was not involved in any other study-related activities, was responsible for handling the investigational products at the study site. The treatment phase was conducted in a double-blinded manner. Although a formal blinding survey was not conducted, all parties, including investigators, participants, site staff, the Contract Research Organization team, study monitors, laboratory personnel, sponsor representatives involved in study oversight, and other designated individuals involved in the conduct of the study, remained blinded to treatment assignments until the study blind was broken following database lock.

### Test Device

Litemed’s pain management device consists of a pair of disk-shaped devices with adhesive patches designed for application on the skin and an orthopedic band meant to secure the devices in place.

Each device has a diameter of 48.6 mm and an exposure area of 18.54 cm², containing 7 LEDs, of which 3 LEDs emit light in the visible red spectrum (660 nm) and 4 LEDs emit light in the infrared spectrum (850 nm). CURAPOD is meant to be used as a pair, doubling the number of LEDs and the area exposed.

The fully charged device is placed on the identified site of pain with the help of the adhesive patches provided and secured in place by wrapping the orthopedic band. It has an autoshutdown mechanism at 30 minutes from activation, following which the orthopedic band is removed and the adhesive is slowly peeled from the skin.

### Placebo

The placebo, identical in form and use to the experimental device, contains visible LEDs that emit visible red light of 0.8 mW irradiance, mimicking the visual experience of the active device without the infrared component.

Both the active and sham devices emitted visible red light to maintain visual similarity during treatment. The active device additionally delivered infrared wavelengths and a higher total energy dose, which were not visually perceptible. A formal assessment of blinding success using postintervention questionnaires was not conducted; therefore, inadvertent unblinding of participants or study personnel cannot be excluded.

Each participant was involved in the study for 1 day, which included 1 hospital visit and 2 telephone calls across 2 time windows. Prospective participants were identified in the outpatient department and inpatient department if they presented with musculoskeletal pain and were screened for eligibility after obtaining informed consent and collecting relevant demographic and medical history data. Treatment was administered according to the randomization schedule after noting the baseline pain intensity, which was measured using the VAS according to the Initiative on Methods, Measurement, and Pain Assessment in Clinical Trials (IMMPACT) guidelines as the responsive primary outcome for acute pain relief, and skin type was assessed using the Fitzpatrick skin type classification [20]. Posttreatment measurements were made 30 minutes after a single session to provide a rest period during which the participants were closely monitored for any adverse events. Participants were handed patient diaries and prescribed rescue medication with relevant instructions, and the follow-up was conducted at the 8- to 12-hour and 21‐ to 24-hour time windows.

### Statistical Analysis

The statistical analysis was computed using SAS software (version 9.4) and used repeated measures ANOVA (RM-ANOVA). Continuous variables were summarized with mean, SD, median, minimum, maximum, and number of nonmissing observations for each treatment group. Categorical variables were summarized by counts and the percentage of participants in corresponding categories. Missing data were minimal and handled using complete case analysis.

An RM-ANOVA was used to assess the varying effectiveness of therapy in terms of changes in VAS scores before and after treatment in both groups at set time points. The analysis focused on the difference in changes in pain scores over time between treatment and placebo groups on various pain sites and skin types.

The primary analysis was performed on the intent-to-treat population and the per-protocol population to assess the difference in treatment efficacy, as measured by the change in VAS score from baseline, between Litemed’s pain management device and the control device.

The secondary analysis was also performed on the intent-to-treat and per-protocol populations. Proportions and percentages were used to compare pain relapse between the CURAPOD and control devices. The chi-square test was used to analyze the percentage of patients whose pain relapse occurred between 8 to 12 hours and 21 to 24 hours.

Analysis of covariance was performed as a supportive analysis to compare posttreatment pain intensity between treatment groups at 30 minutes. The posttreatment VAS score was treated as the dependent variable, and treatment group and pain site were included as fixed effects, with baseline VAS score as a covariate. Safety evaluation was performed on all randomized patients.

A *P* value of <.05 was considered statistically significant for all analyses.

## Results

### Overview

**Figure 1.**
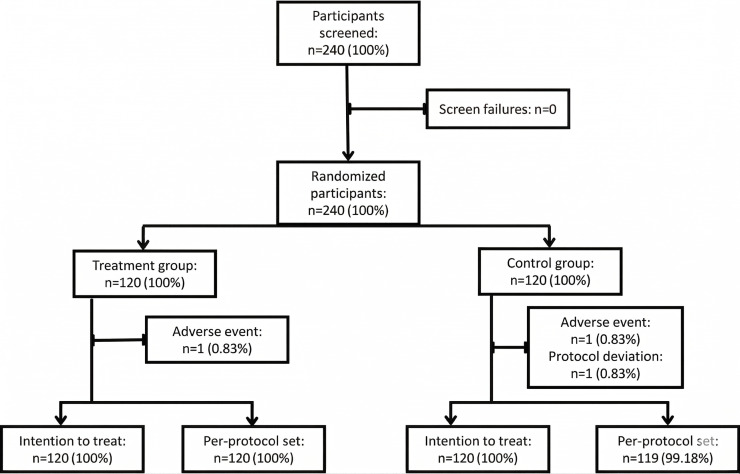
Study flow diagram.

In total, 240 patients were enrolled in the study, of whom 239 (99.5%) completed the study successfully, and 1 protocol deviation was recorded (Figure 1). In total, 10 (4.2%) participants reported receiving 1 dose of rescue medication during the follow-up period; of these, 8 (3.3%) were from the placebo group and 2 (0.8%) were from the treatment group.

### Safety and Tolerability

In total, 2 adverse events were reported (1 in each group). One participant in the placebo arm reported mild irritation at the site of application that resolved spontaneously, and 1 participant from the treatment arm experienced itching and redness at the device application site approximately 15 minutes after application, which resolved after intervention by the study investigator. No serious adverse events or discontinuations due to adverse effects occurred.

### Baseline Characteristics

The 2 study groups were comparable at baseline, with no statistically significant differences in demographic and clinical characteristics as summarized in Table 1.

**Table 1.** Demographic characteristics of study populations at baseline.

Demographic characteristics	Treatment group (n=120), n (%)	Control group (n=120), n (%)	*P* value
Age (years), mean (SD)	38.3 (12.16)	36.5 (12.54)	.25
Age group (years), n (%)			.36
18‐30	38 (31.7)	50 (41.7)	
31‐40	26 (21.7)	21 (17.5)	
41‐50	29 (24.2)	29 (24.2)	
51‐60	27 (22.5)	20 (16.7)	
Sex, n (%)			.90
Female	60 (50.0)	61 (50.8)	
Male	60 (50.0)	59 (49.2)	
Pain condition, n (%)			.61
Acute	61 (50.8)	65 (54.2)	
Chronic	59 (49.2)	55 (45.8)	
Skin type, n (%)			.98
Type 1	10 (8.3)	12 (10.0)	
Type 2	16 (13.3)	17 (14.2)	
Type 3	23 (19.2)	19 (15.8)	
Type 4	27 (22.5)	29 (24.2)	
Type 5	27 (22.5)	25 (20.83)	
Type 6	17 (14.2)	18 (15.0)	

### Descriptive Statistics and Efficacy Outcomes

At baseline, the mean VAS pain score was similar between groups: 5.78 (SD 1.09) in the treatment group and 5.58 (SD 0.95) in the control group. After treatment, greater reductions in pain were observed in the treatment group across all time points evaluated (30 minutes, 8‐12 hours, and 21‐24 hours) compared with the control group, as shown in Table 2.

**Table 2.** Mean VAS^a^ scores, absolute change from baseline, and percentage change for the treatment (n=120) and control (n=120) groups at baseline, 30 minutes, 8–12 hours, and 21–24 hours after treatment. Baseline scores were similar between groups. The treatment group showed greater reductions in pain at all post-treatment time points compared to the control group.

Time point	VAS score, mean (SD)		Change in VAS score, mean (SD)		Percentage change in VAS score, mean (SD)	
	Treatment group (n=120)	Control group (n=120)	Treatment group (n=120)	Control group (n=120)	Treatment group (n=120)	Control group (n=120)
Baseline VAS score	5.78 (1.086)	5.58 (0.949)	—^b^	—	—	—
30 minutes after treatment	2.17 (1.155)	5.38 (1.063)	3.62 (0.963)	0.20 (0.544)	63.64 (16.640)	3.59 (9.439)
8 to 12 hours after treatment	2.06 (1.318)	5.53 (0.987)	3.73 (1.283)	0.06 (0.325)	65.02 (21.216)	1.02 (5.621)
21 to 24 hours after treatment	3.03 (2.037)	5.38 (1.124)	2.75 (2.059)	0.20 (0.740)	47.40 (34.044)	3.31 (12.317)

a VAS: visual analog scale.

b Not applicable.

### Relapses of Pain After Treatment

**Figure 2.**
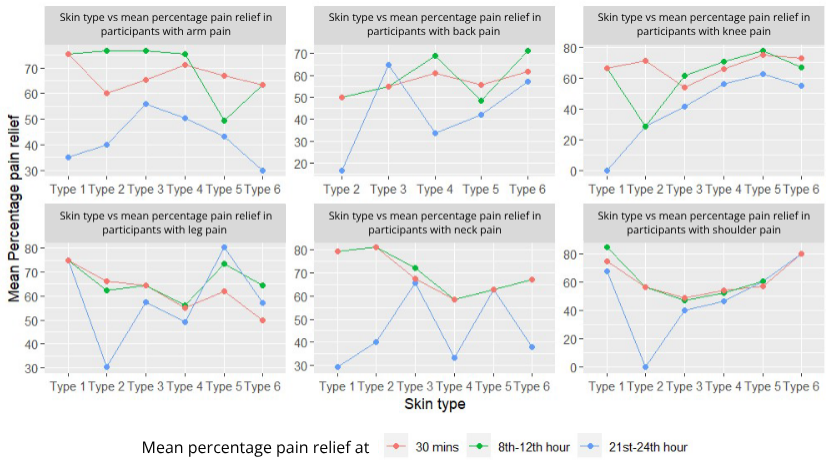
Comparison of mean percentage pain relief of various pain sites across skin types.

Relapse of pain was assessed among the participants who had reported a reduction in pain 30 minutes after treatment, which included 98.3% of (118/120) participants from the treatment group and 14.2% (17/120) of participants from the placebo group. The percentage of relapse of pain is summarized in Figure 2 and Table 3, showing a higher percentage of relapse in the control group.

**Table 3.** Summary of the proportion of participants with pain relapse by treatment arm—intention-to-treat population.

Variable	Treatment group (n=118), n (%)	Control group (n=17), n (%)	*P* value^a^
Telephonic call (status review at 8-12 hours)			<.001
No relapse of pain	110 (91.67)	4 (23.53)	
Pain relapsed	10 (8.33)	13 (76.47)	
Telephonic call (status review at 21-24 hours)			<.001
No relapse of pain	67 (56.78)	1 (5.88)	
Pain relapsed	51 (43.22)	16 (94.12)	

a *P* values were determined using the *χ*2 test.

### RM-ANOVA Analysis

#### Overview

RM-ANOVA was used to evaluate the effect of treatment on VAS scores, controlling for baseline values, pain sites, and skin types.

Table 4 summarizes the descriptive statistics of the change in VAS score, categorized in terms of anatomical site of pain and skin type.

**Table 4.** Descriptive statistics of visual analog scale (VAS) score changes by pain site and skin type in the treatment group.

Subgroup		Control			Treatment		
		Values, n (%)	Mean baseline VAS (SD)	Mean change (SD)	Values, n (%)	Mean baseline VAS (SD)	Mean change (SD)
Pain site							
Arm		20 (16.6)	5.80 (0.83)	0.05 (0.22)	20 (16.6)	5.50 (1.00)	3.65 (0.88)
Back		20 (16.6)	5.45 (0.94)	0.20 (0.70)	20 (16.6)	6.00 (1.08)	3.40 (0.68)
Knee		20 (16.6)	5.50 (1.15)	0.25 (0.55)	20 (16.6)	5.55 (1.15)	3.70 (0.98)
Leg		20 (16.6)	5.65 (1.09)	0.20 (0.52)	20 (16.6)	5.80 (1.24)	3.55 (1.10)
Neck		20 (16.6)	5.45 (0.83)	0.20 (0.70)	20 (16.6)	5.85 (1.09)	3.95 (1.10)
Shoulder		20 (16.6)	5.65 (0.88)	0.30 (0.47)	20 (16.6)	6.00 (0.97)	3.45 (1.00)
Skin type							
Type 1		12 (10)	5.58 (0.67)	0.33 (0.89)	10 (8.3)	5.50 (1.18)	4.10 (0.99)
Type 2		17 (14.1)	5.82 (1.01)	0.12 (0.49)	16 (13.3)	5.81 (0.91)	3.75 (1.24)
Type 3		19 (15.8)	6.00 (1.04)	3.48 (0.79)	23 (19.1)	5.84 (1.01)	0.26 (0.45)
Type 4		29 (24.1)	5.81 (1.08)	3.52 (0.98)	27 (22.5)	5.24 (1.02)	0.21 (0.49)
Type 5		25 (20.8)	5.85 (1.13)	3.67 (1.04)	27 (22.5)	5.44 (0.82)	0.12 (0.33)
Type 6 (skin)		18 (15)	5.47 (1.23)	3.47 (0.72)	17 (14.1)	5.83 (0.92)	0.22 (0.73)

#### Effectiveness of Litemed’s CURAPOD on Change in VAS Score and Pain Site

RM-ANOVA assessing the effects of time, treatment group, and pain site on the change in VAS scores revealed significant main effects of time (*F*_3,684_=282.37; *P*<.001) and treatment group (*F*_1,228_=662.12; *P*<.001), indicating that pain scores changed over time and were significantly lower in the treatment group compared with the control group. However, the effect of pain site was not significant (*F*_5,228_=0.169; *P*=.97), suggesting that the efficacy of the device was consistent across different anatomical locations.

#### Effectiveness of Litemed’s CURAPOD in Pain Relief Among Different Skin Types

All participants were divided into skin types per the Fitzpatrick skin scale at baseline [19]. RM-ANOVA based on the classified skin type revealed no significant effects (*F*_5,228_=0.8; *P*=.55) on change in VAS scores, suggesting that the device is effective across all skin types.

#### Effectiveness of Litemed’s CURAPOD in Pain Relief Among Different Pain Conditions

RM-ANOVA based on pain conditions revealed no significant effects on change in VAS score (*F*_1,236_=1.718; *P*=.19), suggesting that the duration of pain (acute or chronic) does not affect the efficacy of the device.

#### Effect Size Calculation

Effect size analysis comparing the treatment group with the control group and the incidence of pain relapse are summarized in Tables5  6. Effect size analyses demonstrate that the treatment group demonstrated a greater magnitude of pain reduction and relapse prevention. Changes in VAS scores appear to favor the treatment group at all time points, with mean differences of 3.42 (SD 1.12; 95% CI 3.22‐3.62; Cohen *d*=4.33) at 30 minutes, 3.67 (SD 1.46; 95% CI 3.41‐3.93; Cohen *d*=3.40) at 8 to 12 hours, and 2.55 (SD 1.65; 95% CI 2.18‐2.92; Cohen *d*=1.65) at 21 to 24 hours. Correspondingly, pain relapse was lower in the treatment group, with 91.7% remaining relapse free at 8 to 12 hours vs 3.3% in the control group (φ=0.72) and 55.8% vs 0.8% at 21 to 24 hours (φ=0.56).

**Table 5.** Treatment-control effect sizes with 95% CIs.

Time point	Mean difference^a^ (SD) ΔVAS^b^	95% CI	Cohen *d*
30 minutes	3.42 (1.12)	3.22‐3.62	4.33
8 to 12 hours	3.67 (1.46)	3.41‐3.93	3.40
21 to 24 hours	2.55 (2.07)	2.18‐2.92	1.65

a Mean difference is the difference between the treatment and control groups in mean change from baseline VAS score at the specified time point.

b VAS: visual analog scale.

**Table 6.** Relapse outcomes (chi-square test).

Time point	No relapse treatment (n=120), n (%)	No relapse control (n=120), n (%)	φ
8 to 12 hours	110 (91.7)	4 (3.3)	0.72
21 to 24 hours	67 (55.8)	1 (0.8)	0.56

#### Sensitivity Analysis

Sensitivity analysis was performed using analysis of covariance, adjusting for baseline VAS score and pain site. A statistically significant effect of treatment was observed on posttreatment VAS scores at 30 minutes (*F*_1,232_=1180.88; *P*<.0001). The effect of pain site was not statistically significant (*F*_5,232_=0.86; *P*=.51), consistent in direction and magnitude with the primary analysis.

## Discussion

### Principal Findings

This study aimed to evaluate the safety and efficacy of the CURAPOD device, which uses specific wavelengths of light applied to the skin to promote tissue healing and alleviate pain in both acute and chronic conditions.

Participants in the treatment group reported significant reductions in pain scores as early as 30 minutes after treatment, with sustained effects observed at 8 to 12 hours and 21 to 24 hours. The magnitude of change in VAS scores and percentage reduction in pain were consistently higher in the CURAPOD group compared with controls at all time points assessed. The analysis of covariance findings further support the observed treatment effects, demonstrating that the reduction in pain at 30 minutes remained statistically significant after adjustment for baseline pain severity and pain site. However, the magnitude of the observed effect sizes should be interpreted cautiously, as outcomes were assessed immediately following a single treatment session using a highly sensitive and subjective pain scale (VAS), conditions that may accentuate short-term treatment contrasts.

Notably, the frequency of reporting a relapse in pain was significantly lower in the treatment group, suggesting a more durable therapeutic effect. However, relapse rates were assessed only among participants who experienced an initial reduction in pain, leading to a substantial imbalance in denominators (17 in the placebo group vs 118 in the treatment group), possibly distorting the analysis of pain recurrence between the 2 groups. Additionally, as no formal blinding assessment was performed, inadvertent blinding may have occurred, resulting in the vast differences in relapse rates between the 2 groups.

As the depth of light penetration is influenced by both wavelength and the melanin concentration in the skin, participants were categorized using the Fitzpatrick scale to evaluate the device’s efficacy across different skin types [21]. The results showed that participants in the treatment group reported a significant reduction in pain after treatment, irrespective of their skin type, suggesting that CURAPOD’s effect is consistent across diverse skin tones. Other LED-based photobiomodulation devices have also shown promise in clinical studies for conditions such as knee pain and chronic lower back pain, with reported benefits including reduced pain intensity and decreased reliance on analgesic medications [22-26]. Our findings position Litemed’s CURAPOD as a viable adjunct to pain management, delivering pain relief within 30 minutes of first use across diverse pain sites and skin types. The relatively small sample sizes of the subgroups (ie, skin types and pain site) and the geographical restriction of study sites to South India may affect the precision of subgroup analysis and must be taken into consideration when interpreting the generalizability of our findings.

This clinical study demonstrated the safety and efficacy of CURAPOD in this study population within the constraints of a short-term randomized trial, showing reduced pain and a lower proportion of relapse within 24 hours compared with the control device. However, this does not translate to clinical efficacy of the device, and our findings should be considered in view of the several limitations of this study. The protocol limited treatment to one 30-minute session, which is insufficient to establish sustained or cumulative long-term efficacy across various pain conditions. RM-ANOVA showed time effects but was limited due to the nature and duration of follow-up, which lasted a maximum of 24 hours. Telephonic follow-up renders the outcome vulnerable to recall bias, nonresponse (as seen with 1 participant who was lost to follow-up), or interviewer influence. Most importantly, efficacy assessments were based on self-reported VAS scores. Our single-session study design precluded the use of accompanying objective measures such as range of motion, functional activity levels, biomarker analysis (eg, inflammatory cytokines), or neuroimaging such as functional magnetic resonance imaging to monitor and validate pain reduction. Reliance on subjective measures alone may have contributed to information bias and potential placebo effects that were amplified by the device’s heat sensation. An official blinding validation was not conducted; while the treatment and placebo devices both emitted visible red light for visual similarity, sensory matching was not carried out, which may have inadvertently led to partial unblinding. The sensory stimulation from the active device may cause counter-stimulation, which can distract from pain independently of the therapeutic effects of the device and is likely to have contributed to the large effect size observed in our study. These limitations need to be taken into consideration while planning future studies with long-term follow-ups to include objective measures of pain and blinding validation to accurately assess the efficacy of the device in pain management.

### Conclusions

Within the constraints of this study, the CURAPOD device was associated with greater short-term reductions in self-reported musculoskeletal pain compared with sham treatment. Litemed’s CURAPOD device was associated with pain reduction (up to 60% within 30 minutes) for acute and chronic musculoskeletal pain across various sites and Fitzpatrick skin types, as observed in this single-session, double-blind randomized controlled trial. Participants reported relief sustained up to 24 hours, with lower relapse rates compared with placebo, along with an excellent safety profile and no serious adverse events. However, these findings must be interpreted considering study limitations, including short-term follow-up, reliance on subjective VAS measures, and regional sampling, which preclude conclusions about long-term efficacy or broad generalizability. Future multisession trials with objective outcomes in diverse populations are required to further validate CURAPOD’s therapeutic potential as an adjunct for acute pain management in clinical practice.

## Supplementary material

10.2196/87566 Checklist 1 CONSORT-EHEALTH checklist.

## Abbreviations

IMMPACT: Initiative on Methods, Measurement, and Pain Assessment in Clinical Trials
RM-ANOVA: repeated measures ANOVA
VAS: visual analog scale
