# Supplementary material for: Assessing the Safety and Efficacy of a Noninvasive Device in the Management of Musculoskeletal Pain Using Low-Level Light Therapy: Double-Blinded, Randomized, Placebo-Controlled, Multicentric Study
Source: JMIR Biomed Eng. 2026 Apr 16;11:e87566. doi: 10.2196/87566 (PMC13086183; doi:10.2196/87566)
Supplement: Checklist 1 [file biomedeng-v11-e87566-s001.pdf]

CONSORT-EHEALTH (V 1.6.1)

Submission/Publication Form

For Randomized Controlled Trials Evaluating Web-Based and Internet-Based Interventions

---

INSTRUCTIONS

The CONSORT-EHEALTH checklist is intended for authors of randomized trials evaluating web-based and Internet-based applications/interventions, including mobile interventions, electronic games (multiplayer games), social media, telehealth applications, and other interactive and networked electronic applications.

Purpose of Checklist

The CONSORT-EHEALTH checklist and guideline serves two primary purposes:

a) **As a guide for reporting** – Authors of RCTs should use this as a framework for comprehensive manuscript reporting

b) **As a basis for appraisal** – It forms the foundation for evaluating the validity and applicability of eHealth trials

Item Classification

- **Items numbered 1, 2, 3, 4a, 4b, etc.** – Original CONSORT or CONSORT-NPT (non-pharmacologic treatment) items
- **Items with Roman numerals (i, ii, iii, iv, etc.)** – CONSORT-EHEALTH extensions/clarifications
- **Items marked with red asterisk (*)** – Mandatory reporting items

Importance Rating

You are requested to rate each item on a scale of 1–5 for how important/useful you feel it is for the purpose of the checklist and reporting guideline (optional):

- 1 = Not at all important
- 5 = Essential

Submission Instructions

IMPORTANT: Your answers will be published as a supplementary file in JMIR publications and are considered part of your publication if accepted.

Submission Format:

- Copy and paste relevant sections from your manuscript into text boxes (include quotation marks "like this" for direct quotes)
- Or answer directly by providing additional information not in the manuscript
- Or briefly explain why an item is not applicable to your study
- Use proper spelling, grammar, correct capitalization, and avoid abbreviations

Citation Suggestion

Eysenbach G, CONSORT-EHEALTH Group. CONSORT-EHEALTH: Improving and Standardizing Evaluation Reports of Web-based and Mobile Health Interventions. *J Med Internet Res*. 2011;13(4):e126. doi: 10.2196/jmir.1923 [1]

AUTHOR INFORMATION

Your Name:

Dr. Surya Prakash Maguluri

Primary Affiliation (short), City, Country:

Litemed India Pvt Ltd, Telangana, India

Your E-mail Address:

clinicalresearch@litemed.co.in

Title of Your Manuscript:

Assessing the Safety and Efficacy of a non-invasive device in the management of musculoskeletal pain using low-level light therapy: A double-blinded, randomized, placebo-controlled, multicentric study

Name of Your App/Software/Intervention:

CURAPOD

Evaluated Version (if any): Version 1

Language(s) of Intervention:

NA

URL of Intervention Website or App:

https://litemed.co.in/

URL of Image/Screenshot (optional):

Accessibility:

- ☐ Access is free and open
- ☐ Access only for special usergroups, not open
- ☐ Access is open to everyone, but requires payment/subscription/in-app purchases
- ☐ App/intervention no longer accessible
- ☐ **Other: Portable NIR(Near Infrared Radiation) device used for pain management**

Primary Medical Indication/Disease/Condition:

Musculoskeletal pain

Primary Outcomes Measured in Trial:

- *To evaluate the safety and efficacy of CURAPOD in relieving musculoskeletal pain*
- *To compare the average pain score between the subjects using test and control devices*

Secondary/Other Outcomes:

- *To check the relapse time of the pain*
- *To check the efficacy of the device in different skin type*

Recommended "Dose":

- ☐ **Approximately Daily**
- ☐ Approximately Weekly
- ☐ Approximately Monthly
- ☐ Approximately Yearly
- ☐ "As needed"
- ☐ Other:

Approx. Percentage of Users (starters) Still Using the App as Recommended After 3 Months:

- ☐ **Unknown / not evaluated**
- ☐ 0–10%
- ☐ 11–20%
- ☐ 21–30%
- ☐ 31–40%
- ☐ 41–50%
- ☐ 51–60%
- ☐ 61–70%
- ☐ 71–80%

- ☐ 81–90%
- ☐ 91–100%
- ☐ Other:

Overall, Was the App/Intervention Effective?

- ☐ **<u>Yes – All primary outcomes were significantly better in intervention group vs. control</u>**
- ☐ Partly – Some primary outcomes were significantly better in intervention group vs. control
- ☐ No statistically significant difference between control and intervention
- ☐ Potentially harmful – Control was significantly better than intervention in one or more outcomes
- ☐ Inconclusive – More research is needed
- ☐ Other:

Article Preparation Status/Stage:

- ☐ Not submitted yet – in early draft status
- ☐ Not submitted yet – in late draft status, just before submission
- ☐ **<u>Submitted to a journal but not reviewed yet</u>**
- ☐ Submitted to a journal and after receiving initial reviewer comments
- ☐ Submitted to a journal and accepted, but not published yet
- ☐ Published
- ☐ Other:

Journal Target:

- ☐ Not submitted yet / unclear where I will submit this
- ☐ **<u>Journal of Medical Internet Research (JMIR)</u>**
- ☐ JMIR mHealth and UHealth
- ☐ JMIR Serious Games
- ☐ JMIR Mental Health
- ☐ JMIR Public Health
- ☐ JMIR Formative Research
- ☐ Other JMIR sister journal
- ☐ Other:

Is This a Full Powered Effectiveness Trial or a Pilot/Feasibility Trial?

- ☐ Pilot/feasibility
- ☐ **<u>Fully powered</u>**

Manuscript Tracking Number (if JMIR submission): JBME ms#87566

[The ms tracking number can be found in the submission acknowledgement email, or when you login as author in JMIR. If already published in JMIR, it is the four-digit number at the end of the DOI]

- ☐ No ms number (yet) / not (yet) submitted to / published in JMIR
- ☐ **<u>Other</u>: JBME ms#87566**

SECTION 1: TITLE AND ABSTRACT

1a) TITLE: Identification as a Randomized Trial in the Title

CONSORT Item 1a: Does your paper address CONSORT item 1a?

Does the title contain the phrase "Randomized Controlled Trial"? (If not, explain the reason under "Other")

- ☐ **<u>Yes</u>**
- ☐ Other:

Your Response:

1a-i) Identify the Mode of Delivery in the Title

Importance Rating: ☐ 1 ☐ 2 ☐ 3 ☐ 4 ☐ 5 Essential

Identify the mode of delivery. Preferably use "web-based" and/or "mobile" and/or "electronic game" in the title. Avoid ambiguous terms like "online", "virtual", "interactive". Use "Internet-based" only if intervention includes non-web-based Internet components (e.g., email). Use "computer-based" or "electronic" only if offline products are used. Use "virtual" only in the context of "virtual reality" (3-D worlds). Use "online" only in the context of "online support groups". Complement or substitute product names with broader terms for the class of products.

Does your paper address subitem 1a-i?

Yes. The title suggests that the device uses low level light therapy

---

1a-ii) Non-Web-Based Components or Important Co-Interventions in Title

Importance Rating: ☐ 1 ☐ 2 ☐ 3 ☐ 4 ☐ 5 Essential

Mention non-web-based components or important co-interventions in title, if any (e.g., "with telephone support").

Does your paper address subitem 1a-ii?

No, no co-interventions delivered

1a-iii) Primary Condition or Target Group in the Title

Importance Rating: ☐ 1 ☐ 2 ☐ 3 ☐ 4 ☐ 5 Essential

Mention primary condition or target group in the title, if any (e.g., "for children with Type I Diabetes").

Example: "A Web-based and Mobile Intervention with Telephone Support for Children with Type I Diabetes: Randomized Controlled Trial"

Does your paper address subitem 1a-iii?

Yes

1b) ABSTRACT: Structured Summary of Trial Design, Methods, Results, and Conclusions

NPT Extension: Description of experimental treatment, comparator, care providers, centers, and blinding status.

1b-i) Key Features/Functionalities/Components of the Intervention and Comparator

Importance Rating: ☐ 1 ☐ 2 ☐ 3 ☐ 4 ☐ 5 Essential

Mention key features/functionalities/components of the intervention and comparator in the abstract. If possible, also mention theories and principles used for designing the site. Keep in mind the needs of systematic reviewers and indexers by including important synonyms.

Note: Only report in the abstract what the main paper is reporting.

Does your paper address subitem 1b-i?

Yes

1b-ii) Level of Human Involvement in the METHODS Section of the ABSTRACT

Importance Rating: ☐ 1 ☐ 2 ☐ 3 ☐ 4 ☐ 5 Essential

Clarify the level of human involvement in the abstract (e.g., "fully automated" vs. "therapist/nurse/care provider/physician-assisted"). Mention number and expertise of providers involved, if any.

Does your paper address subitem 1b-ii?

Yes, the device is self administrable

1b-iii) Open vs. Closed, Web-Based vs. Face-to-Face Assessments in the ABSTRACT

Importance Rating: ☐ 1 ☐ 2 ☐ 3 ☐ 4 ☐ 5 Essential

Mention how participants were recruited (online vs. offline). Clarify if this was a purely web-based trial, or there were face-to-face components. Clearly state if outcomes were self-assessed through questionnaires.

Note: Use "blinded" or "unblinded" to indicate the level of blinding instead of "open".

Does your paper address subitem 1b-iii?

Yes. Double Blinded

1b-iv) RESULTS Section in Abstract Must Contain Use Data

Importance Rating: ☐ 1 ☐ 2 ☐ 3 ☐ 4 ☐ 5 Essential

Report number of participants enrolled/assessed in each group, the use/uptake of the intervention (e.g., attrition/adherence metrics, use over time, number of logins), in addition to primary/secondary outcomes.

Does your paper address subitem 1b-iv?

Yes.

1b-v) CONCLUSIONS/DISCUSSION in Abstract for Negative Trials

Importance Rating: ☐ 1 ☐ 2 ☐ 3 ☐ 4 ☐ 5 Essential

For negative trials: Discuss the primary outcome. If the trial is negative (primary outcome not changed) and the intervention was not used, discuss whether negative results are attributable to lack of uptake and discuss reasons.

Does your paper address subitem 1b-v?

Trial had positive outcome

SECTION 2: INTRODUCTION

2a) Scientific Background and Explanation of Rationale

2a-i) Problem and the Type of System/Solution

Importance Rating: ☐ 1 ☐ 2 ☐ 3 ☐ 4 ☐ 5 Essential

Describe the problem and the type of system/solution that is the object of the study:

- Intended as stand-alone intervention vs. incorporated in broader health care program?
- Intended for a particular patient population?
- Goals of the intervention (e.g., being more cost-effective, replacing or complementing other solutions)?

Does your paper address subitem 2a-i?

Yes

2a-ii) Scientific Background, Rationale: What Is Known About the (Type of) System

Importance Rating: ☐ 1 ☐ 2 ☐ 3 ☐ 4 ☐ 5 Essential

What is known about the (type of) system that is the object of the study? (Discuss the use of similar systems for other conditions/diagnoses, if appropriate.)

Motivation for the study: What are the reasons for and what is the context for this specific study? From which stakeholder viewpoint is the study performed? What is the potential impact of findings?

Briefly justify the choice of the comparator.

Does your paper address subitem 2a-ii?

Yes

2b) Specific Objectives or Hypotheses

CONSORT Item 2b

Does your paper address CONSORT subitem 2b?

The paper describes the two primary and secondary objectives that were tested using the device against a placebo in two separate treatment groups

---

SECTION 3: METHODS

3a) Description of Trial Design

CONSORT Item 3a

Description of trial design (such as parallel, factorial) including allocation ratio.

Does your paper address CONSORT subitem 3a?

The study was a double-blinded, randomized, placebo controlled trial that assessed the efficacy of the device after one visit. Although the article does not explicitly state it, the design is parallel

---

3b) Important Changes to Methods After Trial Commencement

CONSORT Item 3b

Important changes to methods after trial commencement (such as eligibility criteria), with reasons.

Does your paper address CONSORT subitem 3b?

No changes were made

3b-i) Bug Fixes, Downtimes, Content Changes

Importance Rating: ☐ 1 ☐ 2 ☐ 3 ☐ 4 ☐ 5 Essential

eHealth systems are often dynamic systems. Describe important changes made on the intervention or comparator during the trial (e.g., major bug fixes or changes in functionality or content) and other unexpected events that may have influenced study design such as staff changes, system failures/downtimes, etc.

Does your paper address subitem 3b-i?

No changes were made during the trial

4a) Eligibility Criteria for Participants

CONSORT Item 4a

Does your paper address CONSORT subitem 4a?

Yes

4a-i) Computer/Internet Literacy

Importance Rating: ☐ 1 ☐ 2 ☐ 3 ☐ 4 ☐ 5 Essential

Computer/Internet literacy is often an implicit "de facto" eligibility criterion – this should be explicitly clarified.

Does your paper address subitem 4a-i?

Our intervention does not require internet literacy

4a-ii) Open vs. Closed, Web-Based vs. Face-to-Face Assessments

Importance Rating: ☐ 1 ☐ 2 ☐ 3 ☐ 4 ☐ 5 Essential

Mention how participants were recruited (online vs. offline) and clarify if this was a purely web-based trial, or there were face-to-face components. In online-only trials, clarify if participants were quasi-anonymous and whether technical or logistical measures (e.g., cookies, email confirmation, phone calls) were used to detect/prevent multiple identities.

Does your paper address subitem 4a-ii?

Yes. It is an offline trial with a telephonic follow-up

4a-iii) Information Giving During Recruitment

Importance Rating: ☐ 1 ☐ 2 ☐ 3 ☐ 4 ☐ 5 Essential

Specify how participants were briefed for recruitment and in the informed consent procedures (e.g., publish the informed consent documentation as appendix), as this information may have an effect on user self-selection, user expectation, and may also bias results.

Does your paper address subitem 4a-iii?

Our paper states that informed consent was obtained.

4b) Settings and Locations Where the Data Were Collected

CONSORT Item 4b

Does your paper address CONSORT subitem 4b?

Yes

4b-i) Report If Outcomes Were (Self-)Assessed Through Online Questionnaires

Importance Rating: ☐ 1 ☐ 2 ☐ 3 ☐ 4 ☐ 5 Essential

Clearly report if outcomes were (self-)assessed through online questionnaires (as common in web-based trials) or otherwise.

Does your paper address subitem 4b-i?

No. Telephonic follow-up has been described.

4b-ii) Report How Institutional Affiliations Are Displayed

Importance Rating: ☐ 1 ☐ 2 ☐ 3 ☐ 4 ☐ 5 Essential

Report how institutional affiliations are displayed to potential participants on eHealth media, as affiliations with prestigious hospitals or universities may affect volunteer rates, use, and reactions with regards to an intervention.

(Not a required item – describe only if this may bias results)

Does your paper address subitem 4b-ii?

SECTION 4: INTERVENTIONS

5) The Interventions for Each Group with Sufficient Details to Allow Replication

Including how and when they were actually administered.

5-i) Mention Names, Credentials, Affiliations of the Developers, Sponsors, and Owners

Importance Rating: ☐ 1 ☐ 2 ☐ 3 ☐ 4 ☐ 5 Essential

Mention names, credentials, and affiliations of the developers, sponsors, and owners. If authors/evaluators are owners or developers of the software, this needs to be declared in a "Conflict of Interest" section or mentioned elsewhere in the manuscript.

Does your paper address subitem 5-i?

Yes

5-ii) Describe the History/Development Process

Importance Rating: ☐ 1 ☐ 2 ☐ 3 ☐ 4 ☐ 5 Essential

Describe the history/development process of the application and previous formative evaluations (e.g., focus groups, usability testing), as these will have an impact on adoption/use rates and help with interpreting results.

Does your paper address subitem 5-ii?

No

5-iii) Revisions and Updating

Importance Rating: ☐ 1 ☐ 2 ☐ 3 ☐ 4 ☐ 5 Essential

Clearly mention the date and/or version number of the application/intervention (and comparator, if applicable) evaluated. Describe whether the intervention underwent major changes during the evaluation process, or whether the development and/or content was "frozen" during the trial. Describe dynamic components such as news feeds or changing content which may have an impact on the replicability of the intervention.

Does your paper address subitem 5-iii?

No. Not applicable to our device

5-iv) Quality Assurance Methods

Importance Rating: ☐ 1 ☐ 2 ☐ 3 ☐ 4 ☐ 5 Essential

Provide information on quality assurance methods to ensure accuracy and quality of information provided, if applicable.

Does your paper address subitem 5-iv?

No. Not applicable to our device

5-v) Ensure Replicability by Publishing the Source Code

Importance Rating: ☐ 1 ☐ 2 ☐ 3 ☐ 4 ☐ 5 Essential

Ensure replicability by publishing the source code, and/or providing screenshots/screen-capture video, and/or providing flowcharts of the algorithms used. Replicability (i.e., other researchers should in principle be able to replicate the study) is a hallmark of scientific reporting.

Does your paper address subitem 5-v?

No. Not applicable to our device

5-vi) Digital Preservation

Importance Rating: ☐ 1 ☐ 2 ☐ 3 ☐ 4 ☐ 5 Essential

Provide the URL of the application. As the intervention is likely to change or disappear over the course of years, ensure the intervention is archived (Internet Archive, webcitation.org, and/or publishing the source code or screenshots/videos alongside the article). As pages behind login screens cannot be archived, consider creating demo pages which are accessible without login.

Does your paper address subitem 5-vi?

No. Not applicable to our device

5-vii) Access

Importance Rating: ☐ 1 ☐ 2 ☐ 3 ☐ 4 ☐ 5 Essential

Describe how participants accessed the application, in what setting/context, if they had to pay (or were paid) or not, and whether they had to be a member of a specific group. If known, describe how participants obtained "access to the platform and Internet". To ensure access for editors/reviewers/readers, consider providing a "backdoor" login account or demo mode for reviewers/readers to explore the application.

Does your paper address subitem 5-vii?

No. Not applicable to our device

5-viii) Mode of Delivery, Features/Functionalities/Components of the Intervention

Importance Rating: ☐ 1 ☐ 2 ☐ 3 ☐ 4 ☐ 5 Essential

Describe mode of delivery, features/functionalities/components of the intervention and comparator, and the theoretical framework used to design them (instructional strategy, behavior change techniques, persuasive features, etc.). This includes:

- In-depth description of the content (including where it is coming from and who developed it)
- Whether and how it is tailored to individual circumstances and allows users to track their progress and receive feedback
- Description of communication delivery channels and whether communication was synchronous or asynchronous
- Information on presentation strategies, including page design principles, average amount of text on pages, presence of hyperlinks to other resources, etc.

Does your paper address subitem 5-viii?

Not applicable. However, the application of the device and its mechanism of action have been described

---

5-ix) Describe Use Parameters

Importance Rating: ☐ 1 ☐ 2 ☐ 3 ☐ 4 ☐ 5 Essential

Describe use parameters (e.g., intended "doses" and optimal timing for use). Clarify what instructions or recommendations were given to the user regarding timing, frequency, heaviness of use, if any, or was the intervention used ad libitum.

Does your paper address subitem 5-ix?

Yes

5-x) Clarify the Level of Human Involvement

Importance Rating: ☐ 1 ☐ 2 ☐ 3 ☐ 4 ☐ 5 Essential

Clarify the level of human involvement (care providers or health professionals, as well as technical assistance) in the e-intervention or as co-intervention. Detail the number and expertise of professionals involved, if any, as well as type of assistance offered, the timing and frequency of the support, how it is initiated, and the medium by which the assistance is delivered.

It may be necessary to distinguish between the level of human involvement required for the trial, and the level required for a routine application outside of a RCT setting.

Does your paper address subitem 5-x?

Yes. While the intervention was delivered by site staff for the purpose of the study, the device is self-administrable.

5-xi) Report Any Prompts/Reminders Used

Importance Rating: ☐ 1 ☐ 2 ☐ 3 ☐ 4 ☐ 5 Essential

Report any prompts/reminders used: Clarify if there were prompts (letters, emails, phone calls, SMS) to use the application, what triggered them, and frequency. It may be necessary to distinguish between the level of prompts/reminders required for the trial, and the level for a routine application outside of a RCT setting.

Does your paper address subitem 5-xi?

No. Our device can be used as needed

5-xii) Describe Any Co-Interventions (including Training/Support)

Importance Rating: ☐ 1 ☐ 2 ☐ 3 ☐ 4 ☐ 5 Essential

Describe any co-interventions (including training/support): Clearly state any interventions that are provided in addition to the targeted eHealth intervention, as the eHealth intervention may not be designed as a stand-alone intervention. This includes training sessions and support.

It may be necessary to distinguish between the level of training required for the trial, and the level for a routine application outside of a RCT setting.

Does your paper address subitem 5-xii?

No. Not applicable to our device

SECTION 5: OUTCOMES

6a) Completely Defined Pre-Specified Primary and Secondary Outcome Measures

Including how and when they were assessed.

CONSORT Item 6a

Does your paper address CONSORT subitem 6a?

Yes

6a-i) Online Questionnaires: Validation and CHERRIES Criteria

Importance Rating: ☐ 1 ☐ 2 ☐ 3 ☐ 4 ☐ 5 Essential

If outcomes were obtained through online questionnaires, describe if they were validated for online use and apply CHERRIES items to describe how the questionnaires were designed/deployed.

Does your paper address subitem 6a-i?

No. Not applicable to our device

6a-ii) Describe Whether and How "Use" Was Defined/Measured/Monitored

Importance Rating: ☐ 1 ☐ 2 ☐ 3 ☐ 4 ☐ 5 Essential

Describe whether and how "use" (including intensity of use/dosage) was defined/measured/monitored (logins, logfile analysis, etc.). Use/adoption metrics are important process outcomes that should be reported in any eHealth trial.

Does your paper address subitem 6a-ii?

Yes

6a-iii) Describe Whether, How, and When Qualitative Feedback Was Obtained

Importance Rating: ☐ 1 ☐ 2 ☐ 3 ☐ 4 ☐ 5 Essential

Describe whether, how, and when qualitative feedback from participants was obtained (e.g., through emails, feedback forms, interviews, focus groups).

Does your paper address subitem 6a-iii?

Yes

6b) Any Changes to Trial Outcomes After the Trial Commenced

CONSORT Item 6b

Any changes to trial outcomes after the trial commenced, with reasons.

Does your paper address CONSORT subitem 6b?

No changes were made

SECTION 6: SAMPLE SIZE

7a) How Sample Size Was Determined

NPT: When applicable, details of whether and how the clustering by care providers or centers was addressed.

7a-i) Describe Whether and How Expected Attrition Was Taken Into Account

Importance Rating: ☐ 1 ☐ 2 ☐ 3 ☐ 4 ☐ 5 Essential

Describe whether and how expected attrition was taken into account when calculating the sample size.

Does your per address subitem 7a-i?

Not considered

7b) When Applicable, Explanation of Any Interim Analyses and Stopping Guidelines

CONSORT Item 7b

Does your paper address CONSORT subitem 7b?

Not applicable.

SECTION 7: RANDOMIZATION

8a) Method Used to Generate the Random Allocation Sequence

CONSORT Item 8a

NPT: When applicable, how care providers were allocated to each trial group.

Does your paper address CONSORT subitem 8a?

Yes. Randomisation list was (Version 9.4) generated

8b) Type of Randomisation; Details of Any Restriction

CONSORT Item 8b

Type of randomization; details of any restriction (such as blocking and block size).

Does your paper address CONSORT subitem 8b?

No. Study employed stratified block randomization

---

9) Mechanism Used to Implement the Random Allocation Sequence

CONSORT Item 9

Mechanism used to implement the random allocation sequence (such as sequentially numbered containers), describing any steps taken to conceal the sequence until interventions were assigned.

Does your paper address CONSORT subitem 9?

Yes. Randomisation list was generated using SAS version 9.4

---

10) Who Generated the Random Allocation Sequence, Who Enrolled Participants, and Who Assigned Participants to Interventions

CONSORT Item 10

Does your paper address CONSORT subitem 10?

Yes. Randomisation was computer-generated

SECTION 8: BLINDING

11a) If Done, Who Was Blinded After Assignment to Interventions

For example, participants, care providers, those assessing outcomes.

NPT: Whether or not administering co-interventions were blinded to group assignment.

Yes. Study employed a double blinded design.

11a-i) Specify Who Was Blinded, and Who Wasn't

Importance Rating: ☐ 1 ☐ 2 ☐ 3 ☐ 4 ☐ 5 Essential

Specify who was blinded, and who wasn't. Usually, in web-based trials it is not possible to blind the participants (this should be clearly acknowledged), but it may be possible to blind outcome assessors, those doing data analysis, or those administering co-interventions (if any).

Does your paper address subitem 11a-i?

Yes. Subjects, site staff, and Sponsor representatives involved in the study

---

11a-ii) Discuss Whether Participants Knew Which Intervention Was the "Intervention of Interest"

Importance Rating: ☐ 1 ☐ 2 ☐ 3 ☐ 4 ☐ 5 Essential

Informed consent procedures (4a-ii) can create biases and certain expectations. Discuss whether participants knew which intervention was the "intervention of interest" and which one was the "comparator".

Does your paper address subitem 11a-ii?

Yes. Blinding method has been described.

11b) If Relevant, Description of the Similarity of Interventions

CONSORT Item 11b

(This item is usually not relevant for eHealth trials as it refers to similarity of a placebo or sham intervention to an active medication/intervention.)

Does your paper address CONSORT subitem 11b?

Yes. Studies on similar devices have also been cited in the paper.

SECTION 9: STATISTICAL METHODS

12a) Statistical Methods Used to Compare Groups for Primary and Secondary Outcomes

CONSORT Item 12a

NPT: When applicable, details of whether and how the clustering by care providers or centers was addressed.

Does your paper address CONSORT subitem 12a?

Yes

12a-i) Imputation Techniques to Deal With Attrition/Missing Values

Importance Rating: ☐ 1 ☐ 2 ☐ 3 ☐ 4 ☐ 5 Essential

Imputation techniques to deal with attrition/missing values: Not all participants will use the intervention/comparator as intended and attrition is typically high in eHealth trials. Specify how participants who did not use the application or dropped out from the trial were treated in the statistical analysis. A complete case analysis is strongly discouraged, and simple imputation techniques such as LOCF may also be problematic.

Does your paper address subitem 12a-i?

Not addressed

12b) Methods for Additional Analyses

CONSORT Item 12b

Methods for additional analyses, such as subgroup analyses and adjusted analyses.

Does your paper address CONSORT subitem 12b?

Yes.

SECTION 10: ETHICS AND INFORMED CONSENT

X26) REB/IRB Approval and Ethical Considerations

(Recommended as subheading under "Methods" – not a CONSORT item)

X26-i) Comment on Ethics Committee Approval

Importance Rating: ☐ 1 ☐ 2 ☐ 3 ☐ 4 ☐ 5 Essential

Does your paper address subitem X26-i?

Yes

X26-ii) Outline Informed Consent Procedures

Importance Rating: ☐ 1 ☐ 2 ☐ 3 ☐ 4 ☐ 5 Essential

Outline informed consent procedures (e.g., if consent was obtained offline or online – how? Checkbox, etc.?) and what information was provided. See published guidelines for items to be included in informed consent documents.

Does your paper address subitem X26-ii?

Yes, Partially.

X26-iii) Safety and Security Procedures

Importance Rating: ☐ 1 ☐ 2 ☐ 3 ☐ 4 ☐ 5 Essential

Safety and security procedures, including privacy considerations, and any steps taken to reduce the likelihood or detection of harm (e.g., education and training, availability of a hotline).

Does your paper address subitem X26-iii?

Yes

SECTION 11: RESULTS

13a) For Each Group, the Numbers of Participants

For each group, the numbers of participants who were randomly assigned, received intended treatment, and were analyzed for the primary outcome.

CONSORT Item 13a

NPT: The number of care providers or centers performing the intervention in each group and the number of patients treated by each care provider in each center.

Does your paper address CONSORT subitem 13a?

Yes

13b) For Each Group, Losses and Exclusions After Randomisation

CONSORT Item 13b

For each group, losses and exclusions after randomization, together with reasons.

(NOTE: Preferably, this is shown in a CONSORT flow diagram)

Does your paper address CONSORT subitem 13b?

Yes. All participants completed the study

13b-i) Attrition Diagram

Importance Rating: ☐ 1 ☐ 2 ☐ 3 ☐ 4 ☐ 5 Essential

Strongly recommended: An attrition diagram (e.g., proportion of participants still logging in or using the intervention/comparator in each group plotted over time, similar to a survival curve) or other figures or tables demonstrating usage/dose/engagement.

Does your paper address subitem 13b-i?

Yes. Figure 1

14a) Dates Defining the Periods of Recruitment and Follow-Up

CONSORT Item 14a

Does your paper address CONSORT subitem 14a?

Yes, study was conducted over a 3 month period.

14a-i) Indicate If Critical "Secular Events" Fell Into the Study Period

Importance Rating: ☐ 1 ☐ 2 ☐ 3 ☐ 4 ☐ 5 Essential

Indicate if critical "secular events" fell into the study period (e.g., significant changes in Internet resources available or changes in computer hardware or Internet delivery resources).

Does your paper address subitem 14a-i?

No

14b) Why the Trial Ended or Was Stopped (Early)

CONSORT Item 14b

Does your paper address CONSORT subitem 14b?

No. Study was not terminated prematurely

---

15) Baseline Demographic and Clinical Characteristics

CONSORT Item 15

A table showing baseline demographic and clinical characteristics for each group.

NPT: When applicable, a description of care providers (case volume, qualification, expertise, etc.) and centers (volume) in each group.

Does your paper address CONSORT subitem 15?

Yes

15-i) Report Demographics Associated With Digital Divide Issues

Importance Rating: ☐ 1 ☐ 2 ☐ 3 ☐ 4 ☐ 5 Essential

In eHealth trials it is particularly important to report demographics associated with digital divide issues, such as age, education, gender, social-economic status, computer/Internet/eHealth literacy of the participants, if known.

Does your paper address subitem 15-i?

No, not applicable to our study

---

16) For Each Group, Number of Participants (Denominator) Included in Each Analysis

CONSORT Item 16

For each group, number of participants (denominator) included in each analysis and whether the analysis was by original assigned groups.

16-i) Report Multiple "Denominators" and Provide Definitions

Importance Rating: ☐ 1 ☐ 2 ☐ 3 ☐ 4 ☐ 5 Essential

Report multiple "denominators" and provide definitions: Report N's (and effect sizes) "across a range of study participation [and use] thresholds", e.g., N exposed, N consented, N used more than x times, N used more than y weeks, N participants "used" the intervention/comparator at specific pre-defined time points of interest (in absolute and relative numbers per group). Always clearly define "use" of the intervention.

Does your paper address subitem 16-i?

Yes, addressed

SECTION 12: ADDITIONAL INFORMATION

Additional Comments/Information Not Covered Above:

[Space for any additional information not covered above]

REFERENCES

[1] Eysenbach G, CONSORT-EHEALTH Group. CONSORT-EHEALTH: Improving and Standardizing Evaluation Reports of Web-based and Mobile Health Interventions. *J Med Internet Res*. 2011;13(4):e126. doi: 10.2196/jmir.1923. PMID: 22209829. URL: http://www.jmir.org/2011/4/e126/

Document Information

Generated: December 2025

Based on: CONSORT-EHEALTH (V 1.6.1) Submission/Publication Form

For Use With: Randomized Controlled Trials Evaluating Web-Based and Internet-Based Interventions

Important Notes for Completion:

- Answers will be published as a supplementary file to your publication in JMIR and are considered part of your publication if accepted
- Please fill in all questions diligently using proper spelling, grammar, and correct capitalization
- Avoid abbreviations where possible

- Copy and paste relevant manuscript sections where indicated, using quotation marks "like this" for direct quotes
- Alternatively, provide additional information not in the manuscript or explain why items are not applicable
